# When to consider mitral valve surgery in dogs: a comparative review of open mitral valve repair and transcatheter edge-to-edge repair with a proposed selection framework

**DOI:** 10.3389/fvets.2026.1900762

**Published:** 2026-09-17

**Authors:** Sumade Kongchatree, Jatuporn Rattanasrisomporn

**Affiliations:** Department of Companion Animals Clinical Sciences, Faculty of Veterinary Medicine, Kasetsart University, Bangkok, Thailand

**Keywords:** dogs, mitral regurgitation, myxomatous mitral valve disease, open mitral valve repair, TEER, transcatheter edge-to-edge repair, V-Clamp

## Abstract

Myxomatous mitral valve disease (MMVD) is the most common acquired cardiac disease in dogs, and progression to congestive heart failure (CHF) and cardiac death remains common despite optimal medical management. Two surgical interventions are now clinically available for dogs with advanced MMVD: open mitral valve repair (OMVR) under cardiopulmonary bypass and transcatheter edge-to-edge repair (TEER) using the canine V-Clamp device. This narrative review synthesizes the contemporary literature on both techniques and proposes a framework to guide patient selection. OMVR has more than a decade of clinical experience and a published outcome base of more than 1,000 dogs, with surgical survival rates exceeding 99% across all clinical stages and long-term mortality risk modulated by age, body weight, breed, and pulmonary vascular status. V-Clamp TEER has emerged since 2020 with multiple published clinical cohorts demonstrating high acute procedural feasibility (96–97% in the largest series) and, most recently, sustained one-year hemodynamic and remodeling improvements with two-year all-cause survival of 67% at the leading reporting center; outcomes vary across centers, reflecting the interacting influence of patient selection, anatomic eligibility, and operator learning curve. Because the two techniques differ in evidence maturity and in the outcome metrics reported, direct numerical comparison between them requires caution. OMVR achieves more complete mitral regurgitation (MR) reduction, while V-Clamp produces a less invasive double-orifice repair with intermediate hemodynamic effect that may partially regress in the presence of significant residual MR. A proposed selection framework integrates ACVIM stage, mitral valve anatomy, body size, comorbidity burden, and center experience to guide decisions among OMVR, V-Clamp, and continued medical management. Honest owner counseling that includes center-specific experience and the limits of each option remains a clinical priority as the field evolves.

## 1 Introduction

Myxomatous mitral valve disease (MMVD) is the most common acquired cardiac disease in dogs, accounting for more than 70% of cardiovascular diagnoses and affecting the majority of small-breed dogs older than 10 years of age (1, 2). Progressive myxomatous degeneration of the mitral valve leaflets and chordae tendineae produces mitral regurgitation (MR), which drives left atrial and left ventricular volume overload, progressive cardiac remodeling, and ultimately congestive heart failure (CHF) (3). Despite substantial advances in medical management over the past two decades, MMVD remains the leading cause of cardiac death in affected dogs, and optimal pharmacotherapy delays but does not prevent clinical progression (3, 4).

Contemporary medical therapy for MMVD is well characterized. In the preclinical stage with cardiomegaly (ACVIM stage B2), pimobendan delays the onset of CHF or cardiac death by a median of approximately 15 months compared with placebo (4). Once CHF has developed (stage C), combination therapy with a loop diuretic, pimobendan, an angiotensin-converting enzyme inhibitor, and spironolactone produces median survival times of approximately 9 to 12 months (3, 5). These agents prolong life but do not correct the mechanical lesion driving the disease. The mitral valve continues to leak, the left atrium continues to dilate, and every affected dog remains on a trajectory toward refractory CHF, arrhythmia, or cardiac death. An intervention that addresses the mechanical substrate of MMVD, namely the leaking valve itself, is therefore the natural frontier of treatment.

Two surgical options are currently available for dogs with advanced MMVD. Open mitral valve repair (OMVR) under cardiopulmonary bypass, which combines circumferential mitral annuloplasty with artificial chordal replacement, was first reported in its modern form in 2012 and has accumulated more than a decade of clinical experience at specialty centers worldwide (6, 7). Outcomes from the largest published cohort, comprising 1,019 dogs operated at a single Japanese center, demonstrate surgical survival rates exceeding 99% across all ACVIM stages and low long-term mortality risk beyond the early postoperative period (7). Transcatheter edge-to-edge repair (TEER) using the canine-specific V-Clamp device is a more recent development, with the first clinical feasibility report published in 2020 and the first multi-dog prospective clinical cohort in 2024 (8, 9). The V-Clamp approaches the mitral valve through a minimally invasive left thoracotomy and transapical puncture, does not require cardiopulmonary bypass, and produces a double-orifice mitral valve analogous to the surgical Alfieri technique in humans. Published procedural feasibility in the pivotal 50-dog study was 96%, although a subsequent 20-dog retrospective from an independent center reported 65% three-month survival, highlighting the importance of center experience and patient selection (9, 10).

Despite the growing availability of surgical options, clinicians face these choices with limited published guidance. The 2019 American College of Veterinary Internal Medicine consensus guidelines on MMVD acknowledge OMVR as a consideration for advanced-stage patients but provide no detailed patient selection criteria, and predate the V-Clamp clinical literature entirely (3). The only prior narrative review of canine mitral valve surgery focuses exclusively on OMVR techniques from a single group and was published before the pivotal V-Clamp outcome data emerged (11). No published framework currently assists clinicians in deciding whether a given dog is a better candidate for OMVR, TEER, or continued medical management alone, nor in communicating the comparative risks and benefits to owners. As surgical referrals increase and new centers develop V-Clamp programs, this absence of structured selection guidance is an increasingly important clinical gap.

This narrative review synthesizes the contemporary literature on OMVR and V-Clamp TEER in dogs with MMVD. It has three aims. The first is to summarize current published outcomes, complications, and limitations of each technique. The second is to identify the patient, anatomic, and center-specific factors that influence outcomes. The third is to propose a structured framework to guide patient selection and owner counseling (Figure 1). Mitral valve replacement with prosthetic valves, transcatheter mitral valve replacement, and surgical management of congenital mitral dysplasia are outside the scope of this review. Throughout, we attempt to distinguish findings supported by cohort or comparative data from those derived from small case series or expert opinion, and to indicate where current evidence is sufficient to guide practice and where it is not.

**Figure 1:**
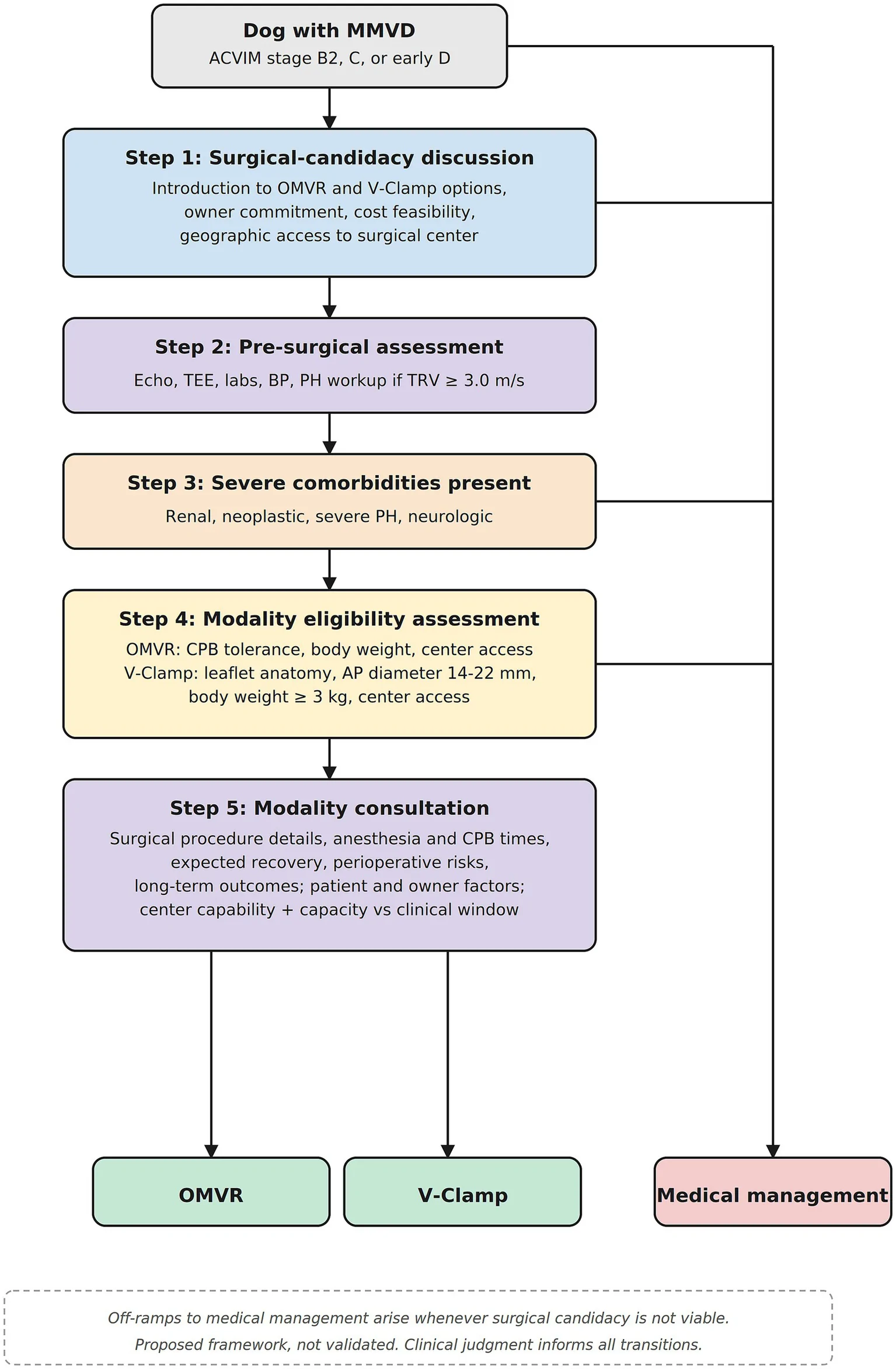
Patient selection framework for canine MMVD surgical referral, illustrating the five-step decision process described in Section 6 and detailed in Table 4. Off-ramps to medical management are available at each step, and an explicit checkpoint for center capability and capacity against the patient’s clinical window is incorporated at Step 5. OMVR, open mitral valve repair; TEER, transcatheter edge-to-edge repair; CPB, cardiopulmonary bypass; AP, anteroposterior; PH, pulmonary hypertension; TRV, tricuspid regurgitant velocity.

### 1.1 Literature identification

This narrative review is based on a structured but non-systematic search of the peer-reviewed literature. We searched PubMed/MEDLINE and Google Scholar from database inception through June 2026, using combinations of the terms “dog” or “canine” with “myxomatous mitral valve disease,” “mitral regurgitation,” “mitral valve repair,” “open mitral valve repair,” “transcatheter edge-to-edge repair,” “V-Clamp,” “TEER,” “cardiopulmonary bypass,” and “pulmonary hypertension.” We supplemented electronic searching by screening the reference lists of retrieved articles and relevant reviews, and by incorporating pivotal human transcatheter mitral repair trials and consensus statements known to the authors. Priority was given to peer-reviewed clinical cohorts and comparative data; single case reports, non-peer-reviewed communications, and conference material were included only where they represented the only available evidence on an emerging topic and are identified as such in the text.

## 2 Pathophysiology and staging context

Myxomatous mitral valve disease is characterized by progressive degeneration of the mitral valve apparatus, with thickening and prolapse of one or both leaflets, elongation or rupture of chordae tendineae, and dilation of the mitral annulus. The resulting mitral regurgitation imposes a chronic volume load on the left atrium and left ventricle, producing eccentric hypertrophy, progressive atrial and ventricular dilation, and eventually elevated left-sided filling pressures (1, 2). Sustained elevation of left atrial pressure transmits to the pulmonary venous circulation and, in a subset of dogs, contributes to pulmonary hypertension (PH) through a combination of passive pressure transmission (postcapillary PH) and, in more advanced disease, pulmonary vascular remodeling (combined pre- and postcapillary PH) (12, 13). Atrial fibrillation, left atrial tear, and cardiogenic pulmonary edema are the principal clinical complications (3).

Three features of this disease are particularly relevant to surgical decision-making. First, the two dominant mechanisms of progressive MR, namely annular dilation and chordal failure (with or without leaflet flail), operate in combination and must both be addressed to produce durable repair (6, 14). Second, left ventricular remodeling in advanced disease produces increased wall stress, myocardial fibrosis, and sometimes irreversible systolic dysfunction, which can limit the reverse remodeling achievable with surgical correction (15, 16). Third, the duration and severity of pulmonary hypertension at the time of surgery influence whether pulmonary vascular pressure will normalize postoperatively or persist as a driver of clinical signs and mortality (17). These three concepts recur throughout the patient selection framework proposed in Section 6.

Clinical staging of MMVD follows the 2019 ACVIM consensus guidelines (3). Stage A denotes dogs at risk for MMVD based on breed but without identifiable disease. Stage B1 denotes dogs with a murmur and echocardiographic evidence of MR but without cardiac enlargement. Stage B2 denotes dogs with echocardiographic or radiographic cardiomegaly meeting established thresholds [left atrium-to-aorta ratio (LA:Ao) at least 1.6, normalized left ventricular internal diameter in diastole (LVIDDN) at least 1.7, vertebral heart score (VHS) greater than 10.5] in the absence of clinical signs. Stage C denotes dogs with current or past clinical signs of heart failure. Stage D denotes refractory CHF despite standard quadruple therapy. Pharmacotherapy is initiated at stage B2 (pimobendan) and intensified progressively through stages C and D (3, 4). In the surgical literature, candidates for mitral valve surgery are predominantly drawn from stages B2, C, and early D, with patient selection criteria varying by technique and center, as discussed in the following sections.

Accurate preoperative staging, echocardiographic assessment of left and right heart size, and quantification of MR severity are prerequisites for rational surgical decision-making. Standardized measurements [LA:Ao, LVIDDN, VHS, vertebral left atrial size (VLAS), tricuspid regurgitant velocity (TRV), and regurgitant fraction (RF)] underpin both the patient selection criteria used by existing surgical programs and the outcomes literature synthesized in this review (18–20).

## 3 Open mitral valve repair (OMVR)

### 3.1 Technique and historical development

Open mitral valve repair for canine MMVD is performed under general anesthesia via a left fifth-intercostal thoracotomy, with the patient placed on cardiopulmonary bypass (CPB) through cannulation of the left carotid artery and the right atrium via the left jugular vein (6, 11). Hypothermic cardioplegic arrest is induced and the left atrium is opened to expose the mitral valve. Repair consists of two principal components performed on the arrested heart: replacement of ruptured or elongated chordae tendineae using expanded polytetrafluoroethylene (ePTFE) sutures, and circumferential annuloplasty to restore normal mitral annular dimensions (6). Following repair, the atriotomy is closed, cardioplegia is reversed with warm reperfusion, and the patient is gradually weaned from CPB. Reported procedure times at experienced centers vary with case complexity; in a recent published cohort, median CPB time was 165 min (range approximately 115 to 260 min) and median total anesthesia time was approximately 7 h (range approximately 4.5 to 10 h), with longer times in more complex cases (7, 17).

The modern canine OMVR technique was first described in its current form by Uechi in 2012, following more than 7 years of development at Nihon University and subsequently at JASMINE Veterinary Cardiovascular Medical Center in Yokohama, Japan (6, 21). The core insight underlying the Uechi technique is that the two dominant mechanisms of MR progression in MMVD, namely annular dilation and chordal failure, operate together and must both be corrected to produce a durable result. Annuloplasty alone fails to address prolapse and flail; chordal replacement alone fails to restore leaflet coaptation against a stretched annulus. Combining the two approaches, each of which has a parallel in human mitral valve surgery, produces a valve that is mechanically competent without the need for prosthetic leaflets or long-term anticoagulation (6). This represents the fundamental advantage of repair over replacement and underlies the durability demonstrated in long-term outcome studies discussed in Section 3.2.

Several technical variations on the Uechi approach have emerged. The loop-in-loop technique, introduced by Matsuura and colleagues at VCA Japan Shiraishi Animal Hospital, uses pre-measured ePTFE loops anchored to a papillary muscle strut to standardize chordal length and potentially shorten aortic cross-clamp time (22). The Chayagasaka group led by Kanemoto has developed an alternative combining artificial chordae with semi-circular suture annuloplasty and direct scallop-suture valvuloplasty for localized leaflet prolapse (11). A novel anchoring technique for artificial chordae, designed to simplify intraoperative decision-making, was reported by Isayama et al. in 2024 (23). Despite these variations, the common therapeutic principle remains consistent: simultaneous correction of annular dilation and chordal failure under cardiopulmonary bypass, using ePTFE as the chordal substitute.

### 3.2 Outcomes

Published outcomes of canine OMVR have evolved from small case series in the early 2010s to multi-100-dog cohorts published in 2022 and 2025, providing increasingly robust estimates of surgical survival, long-term event rates, and predictors of adverse outcome (Table 1).

**Table 1:** Published OMVR outcome studies in dogs with myxomatous mitral valve disease.

Study	Center/country	Cohort size (*n*)	Surgery years	ACVIM stage distribution	Surgical survival/discharge	Postoperative outcomes	Notable findings
Kurogochi et al. (7)	JASMINE, Japan	1,019	2017–2020	B2: 320 (31.4%); C: 562 (55.2%); D: 137 (13.4%)	Surgical survival >99% across all stages; discharge prevalence 95.3% (B2), 95.7% (C), 91.2% (D)	Composite event rate (all-cause mortality + new CHF) at 1,400 days: ~31% (B2), ~34% (C), ~45% (D); incidence 7.1 events per 1,000 dog-months in late period; HR 2.5 with preoperative TRV > 3.7 m/s	Largest published OMVR cohort; documented surgeon learning curve (HR 0.29 in year 4 versus year 1); single-surgeon series; risk factors identified for age, body weight, CKCS breed, TRV > 3.7 m/s
Matsuura et al. (22)	VCA Japan Shiraishi Animal Hospital	55	2017–2020	All Stage C (Stage B2: 14; Stage C: 27; Stage D: 14)	Stage B2: 100%; Stage C: 96.2%; Stage D: 71.4% (log-rank *p* = 0.030)	96% drug-free rate at 3 months postoperative; sustained reverse remodeling at 24 months: LA/Ao < 1.6, LVIDDN <1.7, VHS < 10.6	Loop-in-loop technique for chordal replacement; demonstrated feasibility outside JASMINE; high drug-free rate distinguishes this cohort
Uechi et al. (JAVMA) (21)	Nihon University, Japan	48	2006–2009	Mixed (B2/C/D not separately reported in abstract)	Surgical mortality 17% overall	NR in abstract	First major published OMVR series; established CPB safety in small-breed dogs; foundational paper for canine open mitral valve repair
Yoshida et al. (17)	JASMINE, Japan	21 (PH subset)	Subset of larger JASMINE cohort	C: 12 (57%); D: 9 (43%); all dogs had presumptive group 2 PH preoperatively	Resolution of clinical PH signs in 71.4%; significant postoperative reductions in LA:Ao, LVIDDN, TRV	Median survival 767 days vs. 456-day medical-management benchmark (13); 9.5% residual PH; 14.2% recurrent PH between 6 and 26 months postoperatively	First OMVR cohort focused specifically on PH dogs; identifies ~24% of preoperative PH dogs whose pulmonary vascular disease does not fully reverse with surgical correction of left-sided lesion

ACVIM, American College of Veterinary Internal Medicine; CHF, congestive heart failure; CKCS, Cavalier King Charles Spaniel; CPB, cardiopulmonary bypass; HR, hazard ratio; LA:Ao, left atrial to aortic dimension ratio; LVIDDN, left ventricular internal diameter in diastole normalized to body weight; MMVD, myxomatous mitral valve disease; NR, not reported; OMVR, open mitral valve repair; PH, pulmonary hypertension; TRV, tricuspid regurgitant velocity; VHS, vertebral heart score. These four cohorts span three Japanese specialty centers: Nihon University in Tokyo [Uechi et al. (21), the foundational modern series], VCA Japan Shiraishi Animal Hospital in Saitama [Matsuura et al. (22)], and JASMINE Veterinary Cardiovascular Medical Center in Yokohama [Kurogochi et al. (7); Yoshida et al. (17)]. Uechi et al. (21) founded JASMINE after publishing the 2012 Nihon University paper. Yoshida et al. (17) represents a subset analysis of dogs with preoperative pulmonary hypertension from the larger JASMINE cohort. The geographic concentration of OMVR experience in Japan reflects the procedure’s developmental history; OMVR is now performed at multiple additional centers worldwide (see Section 3.4). Kurogochi et al. (7) used a composite outcome (all-cause mortality + new CHF + second MVR) rather than pure mortality; outcome metrics reflect this composite. “Surgical survival” refers to the proportion of dogs alive at the conclusion of the surgical procedure; “discharge” refers to alive at hospital discharge (typically five days postoperative).

The largest published cohort to date comprises 1,019 dogs with ACVIM stage B2, C, or D MMVD who underwent OMVR at a single Japanese specialty center between 2017 and 2020 (7). Surgical survival, defined as survival of the operative procedure itself, was 99.4% or higher across all clinical stages (stage B2 = 320 of 320, stage C = 560 of 562, stage D = 136 of 137; *p* = 0.78). Survival to hospital discharge was 95.3% for stage B2, 95.7% for stage C, and 91.2% for stage D. Two distinct periods of risk emerged on landmark analysis. In the early postoperative period defined as less than 100 days from surgery, stage D dogs experienced a 2.2-fold higher hazard of death, new CHF, or surgical revision compared with stage B2 dogs [hazard ratio (HR): 2.2; 95% confidence interval (CI): 1.1–4.5]. In the late postoperative period of 100 to 1,400 days, clinical stage was no longer predictive of outcome. Instead, older age (HR 1.3 per year), higher body weight (HR 1.1 per kilogram), Cavalier King Charles Spaniel breed compared with Chihuahua (HR 2.2), and TRV greater than 3.7 m/s (HR 2.5) were independently associated with late events (7). The clinical interpretation is that stage D dogs face elevated perioperative risk, but dogs of any stage who survive the initial postoperative period subsequently share a common long-term prognosis driven primarily by age, body size, breed, and pulmonary vascular status rather than by preoperative cardiac stage.

A parallel cohort of 55 dogs operated at VCA Japan Shiraishi Animal Hospital using the loop-in-loop technique reported stage-specific survival to hospital discharge of 100% for stage B2, 96.2% for stage C, and 71.4% for stage D (22). The survival difference across stages was statistically significant in this smaller cohort (log-rank *p* = 0.030), in contrast to the larger Japanese study. Taken together, these two cohorts suggest that OMVR survival in experienced hands approaches 95 to 100 percent for stage B2 and C dogs, while stage D outcomes vary more widely by center and likely reflect both case selection and team experience. Reverse cardiac remodeling with reductions in left atrial and left ventricular size, VHS, and TRV has been documented in all published cohorts, with most of the remodeling observed within the first 1 to 3 months postoperatively (7, 17, 22). A methodological asymmetry between the OMVR and V-Clamp outcome literatures is worth noting at this point. Published OMVR papers report MR reduction qualitatively (mild/moderate/severe grading or regurgitant jet area to left atrial area ratio), together with reverse-remodeling indices (LA:Ao, LVIDDN, VHS), but typically do not report quantitative postoperative regurgitant fraction (RF) reduction (7, 16, 21, 22). V-Clamp papers, by contrast, consistently report quantitative RF reductions (Section 4.2) (9, 10, 24). This difference reflects the historical reporting conventions of the two literatures rather than a difference in repair quality; OMVR consistently produces more complete MR elimination than V-Clamp, but the magnitude of that reduction has not been reported in the same quantitative units used in the V-Clamp literature. Standardizing the reporting of residual MR severity across techniques is identified as a research priority in Section 7.2.

A particularly important subgroup for clinical decision-making is dogs with preoperative pulmonary hypertension. In a 21-dog cohort of MMVD dogs with presumptive group 2 PH undergoing OMVR, Yoshida and colleagues reported resolution of clinical signs in 71.4% of cases and significant postoperative reductions in LA:Ao, LVIDDN, and TRV (17). However, 9.5% of dogs had residual PH that persisted from surgery, and an additional 14.2% had recurrent PH emerging between 6 and 26 months postoperatively. Median survival time in this cohort was 767 days compared with 456 days reported in medically managed dogs with PH secondary to MMVD (13, 17). These data support OMVR as a survival-extending intervention even in dogs with PH, while identifying an estimated one in four dogs whose pulmonary vascular disease does not fully reverse with surgical correction of the left-sided lesion.

Quality of life outcomes have been less systematically studied but uniformly favorable. Owner-reported quality-of-life scores improve significantly after OMVR and the improvement is sustained for at least 12 months postoperatively (25), with most dogs weaned from preoperative heart failure medications within months of surgery (22). By comparison, dogs managed with optimal medical therapy in the preclinical stage with cardiomegaly (stage B2) have a median time to CHF or cardiac death of approximately 1,228 days on pimobendan (4), and dogs in CHF (stage C) have median survival times of 9 to 12 months with combination therapy (3, 5). The available evidence therefore indicates that OMVR substantially extends life expectancy compared with medical management alone in stages C and D, and is a therapeutic option worth formal consideration from stage B2 onward, with patient-specific factors discussed in detail in Sections 3.3 and 6.

One important caveat applies to this evidence base. Published OMVR outcomes derive predominantly from mature, post-learning-curve experience at high-volume specialty centers, and the early learning-phase data from these programs have not been systematically reported in the peer-reviewed literature. The within-program improvement documented in the Kurogochi 2025 cohort (hazard ratio for postoperative events in year 4 approximately 0.29 relative to year 1) confirms that an institutional learning curve exists for OMVR as well, but the granular early-experience data that have been reported for V-Clamp (e.g., first-half versus second-half cohort comparisons) are not available for OMVR. As a result, direct numeric comparisons of perioperative mortality between OMVR and the more recently introduced V-Clamp TEER (Section 4) should be interpreted with this asymmetric evidence maturity in mind: the OMVR rates reflect mature programs whose early-phase outcomes are unknown, while the V-Clamp rates include both early-program and progressively maturing experience (7).

### 3.3 Complications and limitations

Perioperative mortality after canine OMVR is concentrated in the first 100 days after surgery and is driven by a predictable set of complications. In the largest published cohort, the most frequent causes of early postoperative death were cardiac-related mortality (including low-output syndrome and ventricular arrhythmias), respiratory complications, intraoperative or early postoperative hemorrhage, and neurologic injury (7). Respiratory dysfunction is attributable to both CPB-related lung injury and, less commonly, unilateral diaphragmatic paralysis following thoracotomy (7, 26). Neurologic deficits appear to result from intraoperative cerebral hypoperfusion or postoperative cerebral thromboembolism (7, 11, 27). Interventricular septal hematoma, a recognized complication of canine mitral valve surgery, can produce transient or persistent postoperative ventricular dysfunction (7, 28). Late postoperative events at 100 to 1,400 days are more often driven by non-cardiac causes of death (neoplasia, kidney disease, neurologic disease), reflecting the older median age of surgical candidates (7). Recurring complications across published cohorts include atrial fibrillation both preoperatively and postoperatively (7, 29), device- or suture-related thromboembolism (7), and, as discussed in Section 3.2, residual or recurrent pulmonary hypertension in a subset of dogs with preoperative PH (17). Transesophageal echocardiography is essential for intraoperative guidance, with recent prospective data showing predominantly subclinical esophageal mucosal lesions during probe manipulation (30). Recent work has begun to translate this clinical experience into structured preoperative risk assessment: a recently published prediction model from the same JASMINE cohort identifies preoperative variables associated with 30-day all-cause mortality and provides an open-access risk calculator, offering an early objective tool to inform candidacy discussions and consent (31).

Atrial fibrillation warrants specific consideration, and recent data now inform the OMVR side of this question directly. In a cohort of 28 dogs with preoperative AF undergoing OMVR, spontaneous cardioversion to sinus rhythm occurred in 58% of dogs surviving to the 30-day assessment (14 of 24), consistently at the point of aortic cross-clamp release and without additional electrical or pharmacological cardioversion (29). Thirty-day survival was 86%. Rhythm outcome carried substantial prognostic weight: dogs remaining in persistent AF had a 1,000-day survival of 45% compared with 90% for dogs restoring sinus rhythm (*P* = 0.030), and higher body weight (risk ratio 1.07 per kg) and lower preoperative fractional shortening (risk ratio 0.94 per 1%) predicted persistent AF (29). Even non-converting dogs had a median 30-to-1,000-day survival of 512 days, considerably longer than the approximately 142-day median survival reported for medically managed MMVD dogs with AF that is summarized in the same report (29). The mechanism most consistent with these observations, reverse atrial remodeling following acute annular and volume reduction, is specific to the annuloplasty component of open repair; V-Clamp does not reduce annular dimensions in the same way, and AF-specific outcomes after V-Clamp, including new-onset AF rates, conversion probability, and anticoagulation strategy, are essentially unreported. This asymmetry represents a genuine evidence gap rather than a difference in the procedures themselves, and comparative AF reporting is identified as a research priority in Section 7.2.

Beyond the clinical risks, OMVR is currently limited by three practical constraints. First, the procedure requires cardiopulmonary bypass capability, which in turn requires a dedicated surgical and perfusion team, anesthetic expertise in small and toy breed patients, and postoperative intensive care infrastructure. Worldwide, fewer than 10 centers are known to perform canine OMVR at significant case volume, with the largest concentrated in Japan (6, 7, 11). Second, published outcomes are dominated by single high-volume centers, and it is unclear how readily these results generalize to lower-volume programs; the favorable outcomes reported at JASMINE (Yokohama) and VCA Shiraishi (Saitama) reflect decades of cumulative team experience and a year-over-year reduction in early mortality hazard documented within a single surgeon’s practice (7). Third, the financial cost of OMVR remains substantial for most clients and varies widely by geography, which functions as a de facto access barrier independent of the clinical indications for surgery. These practical limitations matter because they shape the realistic choice set a clinician and owner face when MMVD has progressed to stage C or D, and they are the reason minimally invasive alternatives such as V-Clamp TEER have attracted increasing interest.

### 3.4 Current centers and practical access

Canine OMVR is currently performed at a small number of specialty referral centers worldwide. Historically, the JASMINE Veterinary Cardiovascular Medical Center in Yokohama, Japan, led by Uechi and colleagues, has been the highest-volume program globally, performing several hundred procedures annually (6, 7). Additional established Japanese programs include VCA Japan Shiraishi Animal Hospital in Saitama (Matsuura, now also affiliated with the University of Florida) (22) and the Chayagasaka Animal Hospital in Nagoya (Kanemoto) (11). In the United Kingdom, a dedicated program at the Royal Veterinary College has operated for more than a decade and has contributed to the quality-of-life and complications literature (25, 28). Programs have also been established in South Korea, continental Europe, China, and more recently in the United States, including at the University of Florida and Colorado State University (9, 10). Despite this expansion, the global number of centers performing canine OMVR at significant annual volume remains in the low tens, and most referrals still involve substantial travel and cost for owners. The limited availability of OMVR, combined with its cost and infrastructure requirements, has motivated the development of less resource-intensive surgical alternatives, most prominently V-Clamp transcatheter edge-to-edge repair, which is the subject of the following section.

## 4 V-clamp transcatheter edge-to-edge repair (TEER)

### 4.1 Device and technique

The V-Clamp procedure is performed under general anesthesia through a minimal left thoracotomy in the sixth or seventh intercostal space. Unlike the transseptal approach used in the human MitraClip procedure, in which the device reaches the mitral valve by crossing from the right atrium through the interatrial septum, the canine V-Clamp uses a transapical approach because the smaller cardiac dimensions and vascular anatomy of dogs make transseptal catheter manipulation geometrically challenging. A purse-string suture is placed at the left ventricular apex, through which a 14-French introducer sheath is advanced into the left ventricle and subsequently across the mitral valve into the left atrium under combined transesophageal echocardiography (TEE) and fluoroscopy (9, 10). A mitral crossing device with a soft nitinol basket tip is passed through the introducer to facilitate safe atrial access. The V-Clamp delivery system is then advanced into the left atrium and deployed toward the ventricular side of the mitral valve, where its two arms are positioned across the central segments of the anterior (A2) and posterior (P2) mitral leaflets. The leaflets are grasped between the arms, the clamp is closed and locked, and the delivery system is withdrawn. In cases where residual mitral regurgitation remains significant after deployment of the first clamp, a second clamp can be placed adjacent to the first. The procedure is completed by tying the apical purse-string suture, placing a thoracostomy tube, and closing the thoracotomy. Total procedure time averages approximately 190 min, and dogs are typically discharged within one to 2 days postoperatively (9, 10).

The conceptual foundation of the V-Clamp procedure dates to the late 1990s, when Alfieri and colleagues introduced the surgical “edge-to-edge” technique, in which the free edges of the prolapsing anterior and posterior mitral leaflets are sutured together at the midline to create a double-orifice valve with restored coaptation (32, 33). This surgical concept was subsequently adapted to percutaneous delivery in human cardiology through the MitraClip device, which established transcatheter edge-to-edge repair as a treatment for mitral regurgitation in patients at high or prohibitive surgical risk through the pivotal EVEREST and COAPT trials (34, 35). The canine V-Clamp (Hongyu Medical Technology, Shanghai, China) is a device designed specifically for canine mitral valve anatomy, drawing on the same edge-to-edge principle adapted for the smaller cardiac dimensions and different leaflet geometry of dogs with MMVD (8). Feasibility in healthy dogs was first demonstrated in 2020, and the first prospective clinical cohort in dogs with severe degenerative MR was reported in 2024 (8, 9).

The V-Clamp is available in five sizes with progressively longer clamp lengths and larger arm diameters (14, 16, 18, 20, and most recently 22 mm; the 22 mm Size VI device became commercially available subsequent to the published feasibility cohorts and is now in clinical use at operating centers [B.A. Maran, DVM, DACVIM (Cardiology), Olympic Veterinary Cardiology, personal communication, May 2026]). Size selection is determined by the mid-systolic anterior–posterior (AP) mitral annular diameter measured on preprocedural TEE (9, 10). The most important procedural determinant of durable repair is the extent of leaflet tissue captured within the clamp. A minimum of approximately 4 mm of tissue capture per leaflet is the widely accepted target, verified intraoperatively by measuring residual leaflet length after grasping and comparing it with preprocedural leaflet length (9). Inadequate leaflet capture is associated with the dominant mode of V-Clamp failure, namely single-leaflet device attachment or leaflet tear, as discussed in Section 4.3.

### 4.2 Outcomes

Published outcomes of canine V-Clamp TEER now derive from four primary sources (Table 2): a single-center 50-dog feasibility study from Colorado State University, an extended 111-dog single-institution cohort from the same program with 1- and 2-year follow-up data, a 20-dog single-center retrospective from Korea Animal Medical Center, and a 4-dog communication from Kasetsart University in Thailand (9, 10, 24, 36). Across these four cohorts, reported outcomes span a wide range that, on careful reading, reflects the interacting influence of patient selection, center experience, and anatomic eligibility rather than inherent variability of the device itself.

**Table 2:** Published V-Clamp outcome studies in dogs with myxomatous mitral valve disease.

Study	Center/country	Cohort size (*n*)	Surgery years	ACVIM stage distribution	Procedural success/30-day survival	Postoperative outcomes	Notable findings
Liu et al. (8)	Clinical Medicine Institute, Zhongshan Hospital, Shanghai, China	8	NR	Medication-naive (not on cardiac-specific pharmacotherapy at enrollment); ACVIM staging not explicit but consistent with Stage B1 or early B2 by inclusion criteria	Procedural success 100% (8/8); all dogs survived without complications	Median total surgical procedural time 86.5 min (IQR 76–96.2); median catheterization time 23.5 min (IQR 22–33.8); significant reduction in mitral regurgitant maximum jet area (*p* = 0.012) and jet-area to LA-area ratio (*p* = 0.018)	First reported clinical use of V-Clamp in dogs; established procedural feasibility in canine mitral valve via transapical approach with TEE/fluoroscopic guidance; cohort was medication-naive and not directly comparable to advanced-disease cohorts in subsequent studies; V-Clamp device developed by HongYu Medical Technology, Shanghai
Potter et al. (9)	Colorado State University, United States	50	January 2021–April 2023 (28 months)	Stage B2: 15 (30%); Stage C < 6 mo: 25 (50%); Stage C > 6 mo: 7 (14%); Stage D: 3 (6%); B2 cases added partway through cohort after initial procedural success in Stage C/D	Procedural feasibility 96% (48/50 successful clamp deployment); procedural safety 96% (48/50 discharged from hospital); no procedural deaths; overall procedural success (deployment + discharge) 92%	Median RVol decreased 2.3 → 1.1 mL/kg (*p* < 0.001); median EROA 0.60 → 0.25 cm^2^/m^2^ (*p* < 0.001); median RF 65% → 43% (*p* < 0.001); learning curve documented: mean RF reduction first-half 21.6% ± 28.7%, second-half 53.2% ± 33.4% (*p* = 0.001); device-related adverse event rate 6.3% (3/59 implants), all nonfatal	Pivotal V-Clamp prospective feasibility study; quantifiable learning curve attributed to operator experience AND earlier-stage intervention as cohort progressed; identified anatomic caution criteria (posterior leaflet flail, A-P diameter >20 mm, posterior leaflet length <7 mm, shallow coaptation depth, wide vena contracta); 25 of devices donated by Hongyu Medical Technology; median total procedure time 190 min, median hospitalization 2 days; cohort reflects collective experience of three operators (Orton, Potter, Scansen) rather than a single-operator trajectory
Potter et al. (24)	Colorado State University, United States [extended cohort from Potter et al. (9)]	111	January 2021–January 2025 (4 years)	ACVIM stages B2, C, and D (specific distribution not detailed in available abstract)	Technical feasibility 97.3%; in-hospital mortality 6.3%; 3-month survival 86.4%	Freedom from all-cause mortality: 75% at 1 year, 67% at 2 years. Freedom from cardiac-related mortality: 80% at 1 year, 78% at 2 years. Mean RF 60.6% pre → 35% post → 30.7% at 1 year (sustained MR reduction). Sustained reverse remodeling at 1 year: LVIDdN 2.07 → 1.84; short-axis LA:Ao 2.31 → 2.08. Single clamp 80/108 (74%); two clamps 28/108 (26%)	First peer-reviewed multi-year V-Clamp outcome data; demonstrates that hemodynamic and remodeling improvements achieved in the acute postprocedural window can be sustained at 1 year and that 2-year all-cause survival approaches 67%; substantially extends the V-Clamp evidence base; same institution as Potter 2024 (extended cohort spans 4 years versus the 28-month Potter 2024 cohort); published online ahead of print December 2025 in *Journal of Veterinary Cardiology*
Lee et al. (10)	Korea Animal Medical Center, South Korea	20	September 2023–September 2024 (12 months)	B2: 3 (15%); C: 11 (55%); D: 6 (30%); all dogs receiving pimobendan + furosemide preprocedure	Three-month survival: 65% (13/20) overall; stage-stratified survival B2 100% (3/3), C 63.6% (7/11), D 50% (3/6); 7 deaths within 9 days postoperatively	Mitral leaflet tear/SLDA in four of seven deaths; remaining three deaths from tracheal collapse, presumed post-procedure neurologic deficit (hydrocephalus history), and presumed cardiogenic shock; SLDA attribution: three of four events to challenging anatomy (deep leaflet clefts, thickened leaflets), 1 to operator inexperience. In survivors: VHS 11.6 → 11.0, LA/Ao 2.09 → 1.69, LVIDdN 2.1 → 1.76, RF 77.83% → 55.38%, RVol 3.96 → 1.25 mL/kg at 3 months (significant reverse remodeling)	Independent center counterpoint to Potter cohort; demonstrates V-Clamp outcomes vary by center experience and patient selection; first published V-Clamp outcomes from Korea; high comorbidity burden contributed to mortality; learning curve evident with numerically improved survival between first and second halves of cohort (not statistically significant)
Petchdee et al. (36)	Kasetsart University, Thailand	4	December 2023–March 2024	Two dogs Stage B2; two dogs Stage C	Procedural success in all four dogs (100%); 100% survival to 5–6 months follow-up	Acute MR reduction documented; partial regression of left atrial and ventricular remodeling over months 1 to 5, attributed by authors to residual MR persisting after V-Clamp	First Asian academic-center V-Clamp prospective series; explicit documentation that V-Clamp reverse remodeling can partially regress in the presence of residual MR—strengthens the argument that V-Clamp produces meaningful but often incomplete MR reduction compared to OMVR

ACVIM, American College of Veterinary Internal Medicine; AP, anterior–posterior; EROA, effective regurgitant orifice area; IQR, interquartile range; LA:Ao, left atrial to aortic dimension ratio; LVIDdN, left ventricular internal dimension in diastole normalized to body weight; MR, mitral regurgitation; NR, not reported; RF, regurgitant fraction; RVol, regurgitant volume; SLDA, single-leaflet device attachment; TEE, transesophageal echocardiography; V-Clamp, the canine-specific transcatheter edge-to-edge repair device; VHS, vertebral heart score. Sources: Liu et al. (8); Potter et al. (9); Potter et al. (24); Lee et al. (10); Petchdee et al. (36). The four published V-Clamp cohorts span four geographically independent centers (CSU, Kasetsart, Korea Animal Medical Center, Zhongshan), and the first peer-reviewed multi-year follow-up data have been reported from CSU. Follow-up duration in published V-Clamp series ranges from acute postprocedural assessment [Potter et al. (9)] through 3–6 months [Lee et al. (10); Petchdee et al. (36)] to 1- and 2-year survival data [Potter et al. (24)]; the OMVR evidence base still extends to longer absolute follow-up (≥1,400 days at JASMINE) but the gap between the two evidence bases is narrowing.

The pivotal feasibility study reported by Potter et al. (9) in 2024 included 50 client-owned dogs screened from a surgical referral population over 28 months. The published cohort reflects the collective experience of a multi-operator program at Colorado State University (CSU) led by Orton, Potter, and Scansen, rather than a single-operator trajectory (9). Procedural feasibility, defined as delivery of at least one V-Clamp device, was achieved in 48 of 50 dogs (96%), and survival to discharge was also 96% (9). Among dogs in whom the device was successfully deployed, acute echocardiographic efficacy was substantial: median regurgitant volume fell from 2.3 to 1.1 mL/kg (52% reduction), effective regurgitant orifice area (EROA) fell from 0.60 to 0.25 cm^2^/m^2^ (58% reduction), and RF fell from 65 to 43% (all *p* < 0.001). All reported adverse events were attributed by the authors to operator inexperience or anatomic selection error rather than to device failure, with specific anatomic criteria for patient eligibility discussed in Section 6. A clear learning effect was documented within the study itself, with mean RF reduction improving from 21.6% in the first half of cases to 53.2% in the second half (*p* = 0.001), consistent with human TEER registry data showing continued improvement up to approximately 200 operator cases (9, 37). Initial follow-up in this study was limited to acute postprocedural assessment at 2 to 3 days; longer-term outcomes from this institution have since been reported in an extended cohort.

The longer-term performance of V-Clamp at the same institution has subsequently been reported in an extended cohort by Potter et al. (24). In 111 dogs treated between January 2021 and January 2025, technical feasibility was 97.3%, in-hospital mortality 6.3%, and 3-month survival 86.4%. Freedom from all-cause mortality was 75% at 1 year and 67% at 2 years, and freedom from cardiac-related mortality was 80% at 1 year and 78% at 2 years. Mean regurgitant fraction decreased from 60.6% pre-procedure to 35% post-procedure and was sustained at 30.7% at 1 year, and reductions in normalized left ventricular diastolic dimension and left atrial-to-aortic ratio were similarly maintained at 1 year. These findings provide the first peer-reviewed multi-year outcome data for the V-Clamp device, demonstrate that hemodynamic and remodeling improvements can be sustained beyond the acute postprocedural window, and substantially narrow the gap between V-Clamp and OMVR in terms of evidence maturity, although the longest published OMVR follow-up still extends well beyond the two-year V-Clamp window (7, 24).

A small prospective case series of four dogs operated at Kasetsart University in Thailand between December 2023 and March 2024 reported 100% survival to 5 to 6 months of follow-up, providing the first published V-Clamp outcomes from a Southeast Asian academic program (36). Three of the four dogs were weaned entirely from diuretics and pimobendan. However, echocardiographic reverse remodeling observed in the first 3 days postoperatively partially regressed over months 1 to 5, attributed to residual mitral regurgitation. This finding parallels observations in the human TEER literature where residual MR severity is a strong predictor of long-term outcomes, and introduces the clinically important distinction between acute hemodynamic efficacy and sustained reverse remodeling, which are not equivalent outcomes (35, 38).

The 2025 retrospective by Lee and colleagues reported a materially different outcome profile at an independent Korean center. Of 20 dogs undergoing V-Clamp during the first year of the program’s experience, 13 (65%) survived to 3 months. Seven dogs died within 9 days of the procedure, with mitral leaflet tear or single-leaflet device attachment (SLDA) identified as the mechanism in four of these seven cases (10). Notable features distinguishing this cohort from the Potter cohort include a high proportion of dogs with concurrent comorbidities (including renal, endocrine, and metabolic diseases, with individual cases of tracheal collapse and hydrocephalus reported among the fatal complications), an absence of predefined anatomic exclusion criteria, and three of the four fatal SLDA/leaflet tear cases in which the authors attributed the event to challenging patient anatomy identified preprocedurally (including deep leaflet clefts and markedly thickened leaflets), with the fourth event attributed to operator inexperience. The authors compared outcomes between the first and second halves of the cohort and reported numerically improved survival in the second half, although the difference did not reach statistical significance in this small cohort (10). Echocardiographic reverse remodeling was significant in survivors, indicating that when the device functioned as intended the hemodynamic benefit was real (10).

Synthesizing these three cohorts, the available evidence supports canine V-Clamp TEER as a procedure with high acute technical feasibility in experienced hands and appropriately selected patients, substantial acute reduction in mitral regurgitation severity, and a safety profile highly sensitive to center experience and preprocedural anatomic screening. Prospective multicenter outcome data with follow-up beyond 12 months, standardized anatomic eligibility criteria, and direct comparison to medical management are presently lacking and represent the most important knowledge gaps in the current TEER literature (8–10, 36).

### 4.3 Complications and learning curve

The dominant mode of catastrophic V-Clamp failure is mitral leaflet tear or SLDA, most often occurring within the first several days of the procedure. In the Lee cohort, leaflet tear or SLDA was identified as the mechanism in 4 of 7 early postprocedural deaths; the authors attributed three of these four events to challenging patient anatomy identified preprocedurally (deep leaflet clefts or markedly thickened leaflets), with the fourth event attributed to operator inexperience (10). In the Potter cohort, device-related adverse events occurred in 3 of 59 implanted devices (6.3%), including one case of SLDA, one locking failure, and one locking failure with device embolization to the aorta; all three were nonfatal and were rescued with implantation of a second clamp (9). The principal mechanism common to these events is inadequate leaflet tissue capture, which can result either from insufficient leaflet length at the grasp site, from anatomic features that prevent durable leaflet apposition, or from leaflet tissue fragility that makes the myxomatous leaflet particularly prone to tearing under clamp tension. The reported SLDA rate in canine V-Clamp (up to 20% in the Lee cohort) is broadly comparable to early human MitraClip experience, which reported partial clip detachment in approximately 12.5% of patients in the EVEREST Phase I trial (38), and has decreased substantially at mature high-volume centers as operator experience has accumulated. Contemporary human M-TEER outcomes underscore how much this gap can close: in the EXPAND G4 registry of 1,141 patients treated with the fourth-generation MitraClip device, the 30-day composite major adverse event rate was 2.7%, reflecting the combined effect of device evolution and refined anatomic selection over more than two decades of human experience (39). The comparison illustrates how much room remains for canine V-Clamp outcomes to improve as selection discipline and operator experience accumulate. The persistently elevated rate in current canine V-Clamp series likely reflects the combination of more friable myxomatous leaflet tissue in canine MMVD, less mature operator experience in current canine programs, and the absence of standardized preprocedural anatomic exclusion criteria. Non-fatal complications reported across all three published cohorts include accelerated idioventricular rhythm, which is observed in the majority of cases and is attributed to myocardial injury from the transapical introducer; this arrhythmia is typically self-limiting but occasionally requires short-term lidocaine therapy (9, 10, 36). Device-related or suture-related thrombosis is managed with antiplatelet therapy in all published protocols, although standardization across centers is currently lacking.

The learning curve in canine V-Clamp is quantifiable, clinically meaningful, and has direct implications for owner counseling, with one important interpretive caveat: the published CSU learning curve reflects the cumulative experience of three collaborating operators (Orton, Potter, Scansen) rather than a single operator, which means the institutional curve and any individual operator’s curve are not the same quantity (9). Potter documented a near doubling of echocardiographic efficacy between the first and second halves of the 50-dog cohort (mean RF reduction 21.6 to 53.2%, *p* = 0.001) within a single institution, and Lee documented numerically improved survival between the first and second halves of a 20-dog cohort, although the difference did not reach statistical significance (9, 10). Human MitraClip registry data indicate that procedural outcomes continue to improve up to approximately 200 operator cases, with inflection points previously identified at 50 and 200 cases (37). These data, taken together, support the view that V-Clamp outcomes at any given center should be interpreted in the context of that center’s cumulative experience, that the institutional learning curve described in the published canine V-Clamp literature reflects the program’s collective experience rather than a single operator’s, and that owners being counseled about V-Clamp referral should receive information about both the published literature range and the specific experience of the center performing the procedure.

### 4.4 Current centers and practical access

Canine V-Clamp was developed and first deployed clinically in China, and Hongyu Medical Technology remains the sole manufacturer of the device. Programs performing V-Clamp at meaningful volume have been established in China, South Korea, Thailand, Japan, the United States, and the United Kingdom, with the North American footprint expanding rapidly since 2022 (8–10, 36). In the United States, the program at Colorado State University, led by the group responsible for the pivotal 2024 feasibility study, has served as an early academic anchor, with additional programs now active in multiple regions including the Pacific Northwest (9). Operator training and certification for the V-Clamp device is coordinated through the manufacturer, typically involving didactic instruction, observation of cases at an established center, and supervised initial procedures. This certification pathway differs fundamentally from OMVR, for which surgical training has historically followed informal apprenticeship at established surgical programs rather than a manufacturer-administered credential. The comparatively lower infrastructure burden of V-Clamp, namely the absence of cardiopulmonary bypass requirement, shorter procedure time, and shorter hospitalization, has contributed to its more rapid dissemination relative to OMVR.

## 5 Comparative analysis

Sections 3 and 4 have presented the techniques, outcomes, complications, and global availability of OMVR and V-Clamp TEER separately. The clinical question that follows is how these two options compare on the dimensions that shape individual patient decisions. Figure 2 illustrates the mechanistic contrast between the two repairs. A dimension-by-dimension summary is presented in Table 3 and shown graphically in Figure 3, and this section discusses the interpretation of those comparisons.

**Figure 2:**
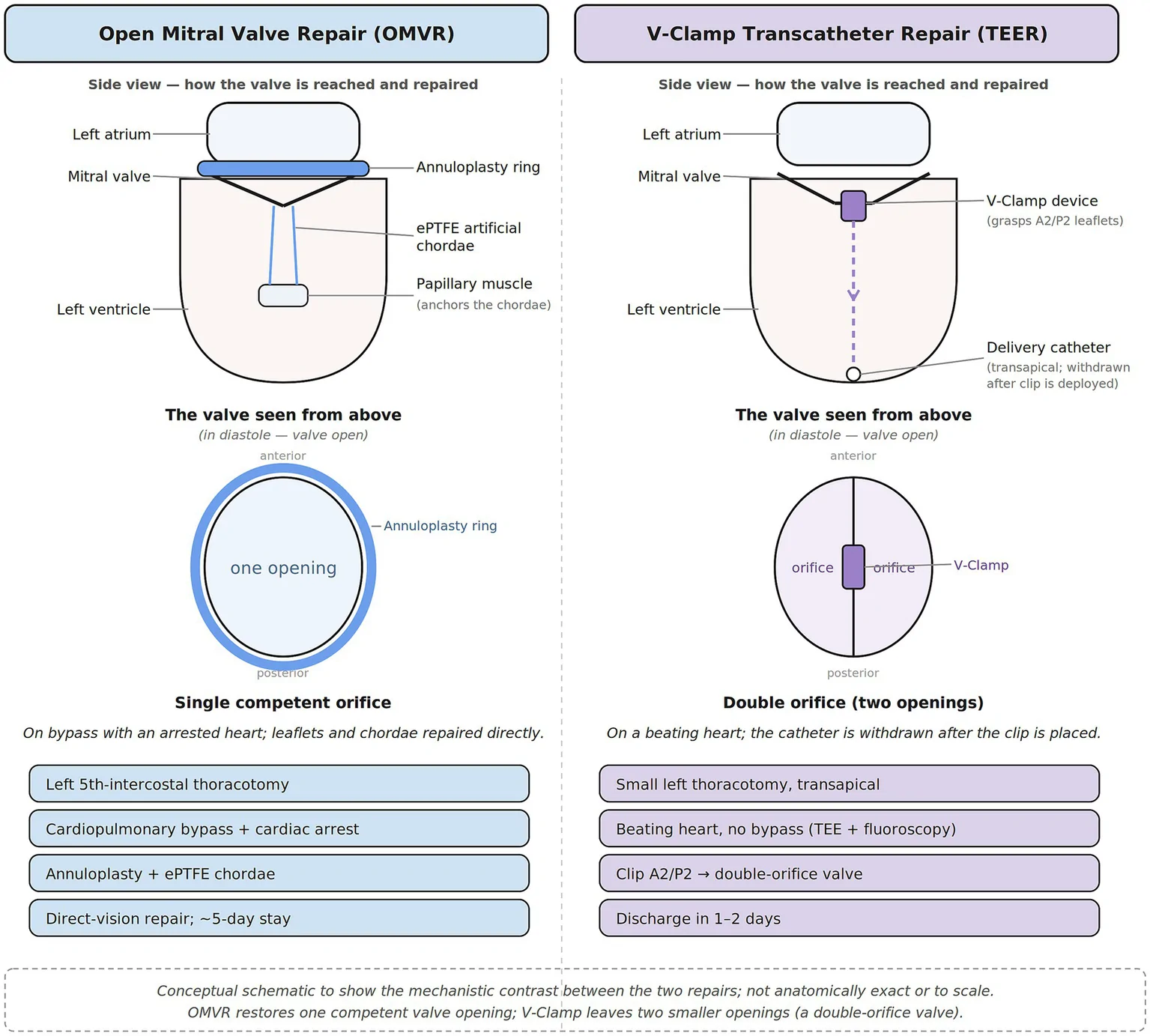
Conceptual comparison of the two surgical approaches. Open mitral valve repair (OMVR, left) is performed under cardiopulmonary bypass on an arrested heart and combines circumferential annuloplasty with ePTFE artificial chordae to restore a single competent orifice. V-Clamp transcatheter edge-to-edge repair (TEER, right) is performed on the beating heart through a transapical catheter under transesophageal echocardiographic and fluoroscopic guidance, clipping the central (A2/P2) leaflet segments to create a double-orifice valve. The schematic is conceptual and is neither anatomically exact nor to scale; it is intended to convey the mechanistic contrast for readers without a surgical background. CPB, cardiopulmonary bypass; ePTFE, expanded polytetrafluoroethylene; TEE, transesophageal echocardiography.

**Table 3:** Comparative summary of OMVR and V-Clamp TEER for canine MMVD.

Dimension	OMVR	V-Clamp TEER
Mechanism of MR reduction	Circumferential mitral annuloplasty + ePTFE chordal replacement; restores leaflet coaptation against a remodeled annulus	Edge-to-edge leaflet apposition at A2/P2 segments creates double-orifice mitral valve, analogous to surgical Alfieri technique in humans
Approach	Left fifth-intercostal thoracotomy under cardiopulmonary bypass with cardiac arrest; surgical valve repair under direct vision	Minimally invasive left lateral thoracotomy (3–4 cm), transapical left ventricular puncture; no CPB; TEE + fluoroscopic guidance
Anatomic eligibility	Broad: applicable to most MMVD anatomies including bileaflet flail, central + non-central segments, larger annular dimensions	Narrower: optimal for A2/P2 prolapse with mid-systolic AP annulus 14–22 mm; cautions noted for posterior leaflet flail, AP diameter >20 mm, posterior leaflet length <7 mm, shallow coaptation depth, wide vena contracta (9)
ACVIM stage candidates in published cohorts	Stages B2, C, and D (with C predominant in largest cohorts; see Table 1)	Stages B2, C, and D (Potter 2024); Stages B2 and C predominant in Lee 2025 and Petchdee 2024 (see Table 2)
Procedure duration	Median total anesthesia ~7 h (range ~4.5–10 h); median CPB time 165 min (range 115–260 min) (Yoshida et al. (17) PH cohort) (17)	Total procedure ~190 min (intubation to extubation); catheterization phase ~25 min [Liu et al. (8); Potter et al. (9)]
Hospitalization	Typically 5 days postoperatively [Kurogochi et al. (7) cohort]	Median 2 days [Potter et al. (9)]
Procedural success/surgical survival	Surgical survival >99% across stages [Kurogochi et al. (7)]; discharge prevalence 91–96% by stage; loop-in-loop cohort 71–100% by stage [Matsuura et al. (22); Kurogochi et al. (7)]	Procedural feasibility 96–97% (Potter cohorts); 65–100% survival to follow-up across published cohorts [Liu et al. (8), Potter et al. (9), Lee et al. (10), Petchdee et al. (36)]
Acute MR reduction	Substantial; reverse remodeling reflected in postoperative VHS, LA:Ao, LVIDDN reductions within 1–3 months (7, 22)	Substantial; median RF 65% → 43%, RVol 2.3 → 1.1 mL/kg, EROA 0.60 → 0.25 cm^2^/m^2^ [Potter et al. (9)]; range 21.6–53.2% RF reduction depending on operator experience (9)
Reverse remodeling at follow-up	Sustained at 1–3 months; medications often discontinuable at 3 months (Matsuura 2022) (22)	Sustained at 1 year in CSU 111-dog cohort (mean RF 30.7%, LVIDdN 2.07 → 1.84) (24); significant remodeling at 3 months in Lee 2025 survivors; partial regression observed over 1–5 months attributed to residual MR [Lee et al. (10); Petchdee et al. (36); Potter et al. (24)]
Long-term outcome data	Multi-year follow-up at 1,400 days; HR-driven analysis of risk factors; mature literature [12 + years since Uechi et al. (21)] (6, 7, 21)	First peer-reviewed multi-year V-Clamp data now available: 1-year all-cause survival 75%, 2-year all-cause survival 67% in 111-dog single-center cohort, with sustained MR reduction at 1 year (mean RF 30.7%) (24); shorter follow-up reported by other centers [3–6 months in Lee et al. (10); Petchdee et al. (36)]; absolute follow-up still substantially shorter than OMVR but rapidly maturing
Catastrophic complications	Cardiac-related death (low-output syndrome, ventricular arrhythmia), respiratory complications (CPB-related lung injury, diaphragmatic paralysis), hemorrhage, neurologic injury, interventricular septal hematoma; concentrated in first 100 days (7, 26–28)	Mitral leaflet tear/single-leaflet device attachment (SLDA), reported up to 20% in Lee cohort; locking failure with device embolization documented [Potter et al. (9); Lee et al. (10)]
Non-fatal complications	Atrial fibrillation, residual or recurrent PH in subset of preoperative PH dogs, postoperative cerebral thromboembolism risk, TEE-related esophageal lesions (mostly subclinical) (7, 17, 30)	Accelerated idioventricular rhythm (most cases, self-limiting), transient atrial fibrillation, postprocedural ventricular ectopy, device thrombosis (managed with antiplatelet therapy) (9, 10, 36)
Atrial fibrillation & antithrombotic management	Preoperative AF converts spontaneously to sinus rhythm in ~58% at cross-clamp release; persistent postoperative AF predicts worse long-term survival (45% vs. 90% at 1,000 days); antithrombotic/antiplatelet therapy per center protocol (7, 29)	New-onset AF rate, conversion probability, and anticoagulation strategy not systematically reported; antiplatelet therapy used for device thromboprophylaxis without cross-center standardization (9, 10, 36)
Center experience required	Highly experienced multi-disciplinary team (surgeon + perfusionist + anesthesiologist + ICU); learning curve documented over hundreds of cases (Kurogochi: HR 0.29 in year 4 vs. year 1) (7)	Significant learning curve at 50 and 200 cases per human MitraClip data; in canine V-Clamp, second-half cohort efficacy approximately doubles compared to first-half (Potter); survival improvement also documented between cohort halves (Lee) (9, 10, 37)
Global availability	<10 high-volume centers worldwide; concentrated historically in Japan (Nihon University, JASMINE Yokohama, VCA Japan Saitama); newer programs in United Kingdom (RVC), United States (UF, CSU), continental Europe, Korea, China (6, 7, 11)	Multiple centers across United States, China, Korea, Thailand, Australia, others; rapidly expanding; concentrated initially at CSU, now adopted by additional centers (9, 10, 36)
Owner counseling considerations	Long surgical day; higher upfront perioperative risk; durable result with mature long-term data; frequent medication discontinuation	Shorter procedure; quick recovery; emerging long-term data with promising 1- and 2-year results from leading center; ongoing learning curves at most centers; outcome quality center-dependent

AP, anterior–posterior; CPB, cardiopulmonary bypass; ePTFE, expanded polytetrafluoroethylene; EROA, effective regurgitant orifice area; HR, hazard ratio; LA:Ao, left atrial to aortic dimension ratio; LVIDDN, left ventricular internal diameter in diastole normalized to body weight; MMVD, myxomatous mitral valve disease; MR, mitral regurgitation; OMVR, open mitral valve repair; PH, pulmonary hypertension; RF, regurgitant fraction; RVol, regurgitant volume; SLDA, single-leaflet device attachment; TEE, transesophageal echocardiography; TEER, transcatheter edge-to-edge repair; VHS, vertebral heart score. All comparative ranges in this table are derived from the published cohorts summarized in Tables 1, 2; specific values should be verified against the cited source for individual decision-making. Direct head-to-head comparison between OMVR and V-Clamp has not been performed in dogs; all comparisons in this table are based on cross-cohort literature synthesis with attendant limitations of selection and center heterogeneity. Procedural success metrics differ between procedures: OMVR papers typically report “surgical survival” or “discharge prevalence,” while V-Clamp papers report “procedural feasibility” or “30-day survival.” These are not directly equivalent metrics and should not be compared as if they were. The OMVR evidence base spans more than a decade; the V-Clamp evidence base spans approximately 5 years from Liu et al. (8) to current, with 1- and 2-year survival data now available from the leading reporting center.

**Figure 3:**
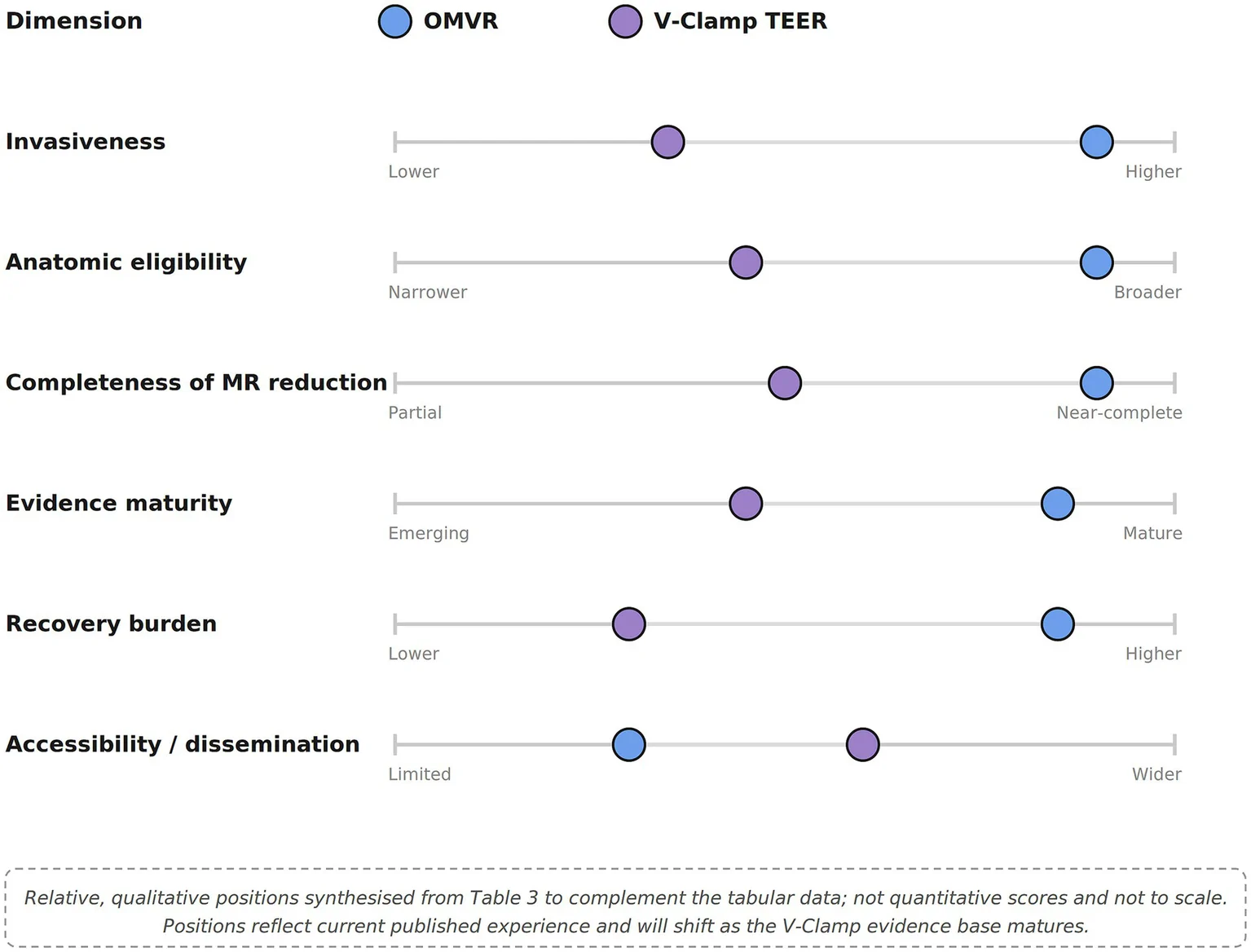
Graphical comparison of OMVR and V-Clamp TEER across key dimensions, complementing Table 3. Positions are relative, qualitative syntheses of the published data rather than quantitative scores, and are not to scale; they reflect current published experience and are expected to shift as the V-Clamp evidence base matures. OMVR, open mitral valve repair; TEER, transcatheter edge-to-edge repair; MR, mitral regurgitation.

### 5.1 Procedural invasiveness and hemodynamic impact

The two procedures differ fundamentally in their physiological burden. OMVR requires sternotomy-free left thoracotomy, cardiopulmonary bypass with hypothermic cardioplegic arrest, and aortic cross-clamping, producing a systemic inflammatory response and a recovery period that includes 4 to 7 h of anesthesia and 5 to 7 days of hospitalization at experienced centers. V-Clamp requires a smaller left thoracotomy, a beating-heart transapical approach, and does not require CPB, with a typical procedure time of approximately 190 min and hospital discharge within 1 to 2 days (7, 9). For frail patients or those with high anesthetic risk, this difference in physiological burden is clinically meaningful. For robust patients with fewer comorbidities, the difference matters less because both procedures are generally well tolerated by such dogs at experienced centers.

### 5.2 Patient eligibility and anatomic constraints

OMVR is technically feasible across a broad range of canine body weights, with the majority of published cohorts reporting median body weights between 3 and 5 kg and successful operations in dogs as small as 1.5 to 2 kg (6, 7). V-Clamp is constrained by device sizing: the smallest available clamp is 14 mm, which corresponds to a minimum mitral annular AP diameter suitable for deployment. Published V-Clamp cohorts have reported median body weights approximately twice those of published OMVR cohorts, reflecting this anatomic floor (9, 10). In the opposite direction, severe leaflet pathology features such as deep clefts, markedly thickened leaflets, significant flail (particularly posterior flail), and inadequate leaflet coaptation depth disqualify candidates from V-Clamp because they predispose to leaflet tear or SLDA, but these same features are not absolute contraindications for OMVR, where they can be addressed directly during repair. OMVR therefore offers wider anatomic eligibility; V-Clamp offers a lower physiological burden in the subset of patients who meet its anatomic criteria.

### 5.3 Outcome profile

OMVR has accumulated more than a decade of outcome data and more than 1,000 published cases at a single center, representing the program’s post-learning-curve experience, with survival to hospital discharge in the mid-90% range for stages B2 and C and long-term event rates driven primarily by age, body weight, breed, and pulmonary vascular status rather than by preoperative stage (7). V-Clamp now has approximately 5 years of published clinical outcome data, with reported procedural success ranging from 65 to 96% across four geographically independent cohorts, and follow-up extending to 1- and 2-year all-cause survival of 75 and 67%, respectively, in the largest reported series (9, 10, 24, 36). The OMVR evidence base is therefore more mature in absolute follow-up duration, but the V-Clamp evidence is rapidly expanding, and the outcome ranges in the published V-Clamp literature are in large part explained by the selection, experience, and anatomic discipline variables discussed in Section 4.3. Direct numeric comparison of perioperative mortality across the two literatures should account for the fact that the published OMVR data describe established programs while V-Clamp data span the field’s first 5 years and include both early-program and progressively maturing experience. More fundamentally, the outcome endpoints themselves are not interchangeable: “surgical survival” (survival of the operative procedure), “survival to discharge,” and “procedural feasibility” (successful device delivery) measure different things, and numerically similar values across these metrics do not indicate clinically equivalent results.

### 5.4 Residual mitral regurgitation

The two techniques produce qualitatively different hemodynamic results. OMVR, when successful, restores near-normal mitral valve competence, with most published cohorts reporting near-complete elimination of MR on post-repair echocardiography. V-Clamp produces a double-orifice valve with an intermediate degree of MR reduction, with reported RF reductions improving from a median of approximately 22% in the first half of the Potter cohort to 53% in the second half, consistent with a within-institution learning effect (9). In the human TEER literature, the magnitude of residual MR after intervention is a strong predictor of long-term outcomes, and Petchdee’s observation that V-Clamp reverse remodeling may partially regress over months 1 to 5 in the presence of residual MR suggests this relationship may hold in dogs as well (35, 36). This difference in hemodynamic endpoint, rather than difference in procedural success, may prove to be the most important long-term distinction between the two techniques.

### 5.5 Cost, access, and evidence maturity

OMVR is performed at fewer than 10 centers worldwide at significant volume and requires infrastructure that limits rapid expansion; V-Clamp is available at a growing number of centers with a shorter training pathway, supporting faster geographic dissemination (6, 7, 9, 10). These structural differences have clinical consequences. A patient for whom OMVR is medically indicated but geographically inaccessible is, in effect, a patient for whom medical management is the only feasible option. A patient for whom V-Clamp is available at a nearby but early-experience center faces a different calculus, in which center-specific outcomes should be weighed explicitly alongside the published literature range.

### 5.6 The cross-cutting variable: center experience

All of the preceding comparisons are modulated by center experience, which affects both techniques but does so differently. OMVR outcomes at JASMINE continued to improve year over year within a single surgeon’s practice even after the center had performed hundreds of operations, suggesting a long continuous learning curve. V-Clamp outcomes improve within the first 50 cases at the center and continue improving, per human MitraClip data, up to approximately 200 cases. At the current state of the field, every V-Clamp program globally is still within its own learning curve. This context should inform how published outcome ranges are applied to individual patient decisions, which is the subject of the patient selection framework in Section 6.

### 5.7 Developmental-stage sensitivity

A comparison matched for programme maturity, rather than for calendar time, places the two techniques in more honest perspective. The earliest published OMVR series reported 17% surgical mortality across its initial 48 cases (21), whereas contemporary post-learning-curve OMVR mortality is below 1% (7). Early-programme V-Clamp experience shows a comparable pattern, with 35% three-month mortality in a first-year cohort that lacked predefined anatomic exclusion criteria (10) against 6.3% in-hospital mortality and 86.4% three-month survival at a mature centre (24). Viewed this way, much of the apparent gap between the two techniques reflects programme maturity and selection discipline rather than intrinsic properties of either procedure, and mature OMVR figures should not be compared with early-programme V-Clamp figures as though they described the same developmental stage.

### 5.8 Postoperative pharmacotherapy

The differing residual MR burden of the two procedures has direct implications for postoperative drug management, a topic addressed only briefly in most primary reports. After OMVR, a high proportion of dogs are weaned from heart failure medication, with one cohort reporting a 96% drug-free rate at 3 months (22). After V-Clamp, the picture is more variable: three of four dogs in one series were weaned entirely from diuretics and pimobendan (36), but the more frequent presence of clinically significant residual MR means that continued therapy is often appropriate. No prospective study has compared weaning strategies after either procedure, so the following criteria are offered as a consensus-free, physiologically grounded starting point rather than as validated guidance: quantified residual MR, normalisation of reverse-remodelling indices (LA:Ao and LVIDDN), absence of congestive signs, and stable renal function and electrolytes, with sequential rather than simultaneous tapering under echocardiographic reassessment, and with pimobendan retained longest where systolic function remains impaired. Antithrombotic protocols after V-Clamp are similarly unstandardised across centres. Standardisation of postoperative pharmacotherapy and antithrombotic protocols after both procedures is identified as a research priority in Section 7.2.

## 6 Proposed patient selection framework

The comparative analysis in Section 5 identifies six dimensions along which OMVR and V-Clamp differ clinically. The proposed framework presented here translates those dimensions into a structured approach for patient selection and owner counseling. Figure 1 presents the framework as a decision tree, and Table 4 summarizes the criteria in tabular form. The framework is intended to support clinical reasoning rather than to function as a validated algorithm, and its elements should be interpreted in the context of individual patient circumstances and evolving published evidence.

**Table 4:** Patient selection framework for canine MMVD surgical referral, paired with Figure 1.

Step	Setting	Key assessments	Criteria favoring continued evaluation	Criteria triggering off-ramp to medical management
Step 1: initial candidacy discussion	Primary care veterinarian (general practice)	Confirm MMVD diagnosis (echocardiography or strong clinical suspicion); discuss owner expectations, finances, and willingness to travel; introduce both OMVR and V-Clamp as available surgical options; raise cost considerations early	ACVIM stage B2 with progressive cardiomegaly, persistently elevated E wave, atrial fibrillation, or atrial rupture (3, 7); ACVIM stage C with first or recurrent CHF episode; ACVIM stage D refractory to maximal medical therapy (3); owner financially and logistically able to pursue advanced care	Owner declines surgical pursuit at any stage (financial, logistical, philosophical); ACVIM stage A or early B1 (no cardiomegaly, no medications); poor general health unrelated to MMVD that would limit benefit
Step 2: pre-surgical assessment	Veterinary cardiologist	Comprehensive echocardiography including LA:Ao, LVIDDN, regurgitant fraction or volume, TEE assessment of mitral anatomy if available; thoracic radiography (VHS); blood pressure; comprehensive bloodwork including renal panel; assessment of pulmonary hypertension status (3, 12)	Confirmed advanced MMVD by echocardiography; preserved or only modestly impaired LV systolic function (preserved fractional shortening); pulmonary hypertension absent or controlled (TRV ≤ 3.7 m/s; severe PH with TRV gradient >80 mmHg is exclusion criterion in published V-Clamp trials) (7, 9, 12); renal function adequate for surgery	Severe pulmonary hypertension with TRV gradient >80 mmHg [V-Clamp exclusion per Potter et al. (9)]; severe end-stage cardiac dysfunction unlikely to recover with intervention; advanced renal failure not amenable to perioperative management
Step 3: comorbidity screening	Cardiologist with general practice or internist input	Screen for non-cardiac contraindications: active systemic infection, immune-mediated disease, neoplasia, severe gastrointestinal/pancreatic disease, severe tracheal collapse, neurologic disease (e.g., hydrocephalus); also assess preoperative azotemia and electrolyte derangements common in advanced MMVD on diuretic therapy (7)	Comorbidities are absent, controlled, or judged unlikely to compromise 6-month survival (9); bloodwork-detected derangements are correctable preoperatively; client able to manage perioperative care	Active systemic infection or immune-mediated disease [V-Clamp exclusion in Potter et al. (9)]; comorbidity judged likely to compromise 6-month survival (9); severe end-stage non-cardiac disease (advanced neoplasia, severe protein-losing enteropathy, etc.)
Step 4: modality eligibility assessment	Cardiologist with surgical-team input or referral	Detailed mitral valve anatomic assessment via TEE focused on segmental involvement, leaflet length, AP annular dimension, leaflet flail, coaptation depth, vena contracta width; assess body weight (V-Clamp device sizes accommodate ~14–22 mm AP annulus with the recent introduction of a Size VI 22 mm device, corresponding to small/medium-breed dogs)	Criteria favoring OMVR: bileaflet flail; A1, A3, P1, or P3 segment involvement; AP annulus >22 mm; posterior leaflet flail; smaller dogs with anatomy outside V-Clamp size range; willingness to accept higher upfront perioperative risk for durable result with mature long-term data (6, 7, 21, 22). Criteria favoring V-Clamp: A2/P2 prolapse with central segment involvement; AP annulus 14–22 mm; posterior leaflet length ≥7 mm; adequate coaptation depth; preference for shorter recovery and lower upfront risk despite emerging long-term data (9, 10, 36)	Anatomy poorly suited to either approach (rare); body size outside V-Clamp range AND OMVR not accessible; severe pulmonary hypertension >80 mmHg not improved with optimization (9)
Step 5: modality consultation and decision	OMVR or V-Clamp surgical team (referral center)	Final review of imaging by surgical team; informed consent discussion regarding center-specific outcomes, perioperative risks, and durability expectations; discussion of operator/team experience at the specific center; coordination of perioperative care including thromboprophylaxis; explicit assessment of center capability and capacity against the patient’s clinical window (7, 9, 10, 36)	Surgical team confirms candidacy and clinic accepts case; owner provides informed consent after reviewing both procedures’ risks and benefits at this center; perioperative coordination feasible with referring practice	Surgical team declines case based on detailed review; owner withdraws after final consultation; logistic or financial barriers emerge that prevent case completion; no center with both adequate capability and available capacity within the patient’s clinical window

ACVIM, American College of Veterinary Internal Medicine; AP, anterior–posterior; CHF, congestive heart failure; LA:Ao, left atrial to aortic dimension ratio; LV, left ventricular; LVIDDN, left ventricular internal diameter in diastole normalized to body weight; MMVD, myxomatous mitral valve disease; OMVR, open mitral valve repair; PH, pulmonary hypertension; TEE, transesophageal echocardiography; TRV, tricuspid regurgitation velocity; VHS, vertebral heart score; V-Clamp, the canine-specific transcatheter edge-to-edge repair device. This framework applies to dogs whose owners have expressed interest in surgical evaluation. It is intended as a structured aid to clinical decision-making, not as rigid eligibility criteria. The framework deliberately introduces both OMVR and V-Clamp early (Step 1) rather than committing to a single modality before specialist evaluation. Final modality selection occurs at Step 4 after full anatomic and clinical assessment, with consultation in Step 5. Cost considerations are introduced at Step 1 (during the primary care discussion) because pursuing the full referral pathway has financial implications at every step. Early transparency about cost protects owners from incurring referral expenses for procedures they ultimately cannot afford. Off-ramps to medical management exist at every step, reflecting the reality that medical management remains a legitimate and clinically appropriate choice at any point in the framework. Specific anatomic criteria for V-Clamp eligibility (Step 4) are based on published case series; these criteria are evolving with growing global experience and may shift as more centers report outcomes. The “AP annulus 14-22 mm” range now reflects the recent introduction of a Size VI 22 mm device by Hongyu Medical Technology (B.A. Maran, personal communication, May 2026), extending beyond the three device sizes (14, 16, 18 mm) used in the Potter 2024 cohort. The framework is intended for use with Figure 1 (decision flowchart). Figure 1 illustrates the sequential structure and off-ramps; Table 4 provides the specific assessment content at each step.

### 6.1 The framework-status note

The framework that follows is intended as a structure for clinical reasoning and transparent owner discussion, not as a validated algorithm. Its categorical form, a decision tree (Figure 1) and a stepwise table (Table 4), should not be read as implying established certainty. In particular, the V-Clamp anatomic eligibility thresholds it incorporates derive from fewer than 200 dogs across all published cohorts, drawn largely from a single centre’s experience, and should be expected to change as additional centres report. A recent review co-authored by the V-Clamp programme’s own leadership and a human transcatheter-valve trialist similarly characterises canine transcatheter edge-to-edge repair as still early on its learning curve (40). The framework should therefore be applied as a reasoning aid, with its individual thresholds treated as provisional.

### 6.2 Selection criteria

#### 6.2.1 ACVIM stage

Surgical consideration is generally reserved for dogs in stages B2, C, and early D. Stage B1 dogs without cardiac enlargement have no established indication for intervention, and stage D dogs who are actively decompensating pose elevated perioperative risk for both techniques (3, 7). Within the surgical-candidate range, stage is less discriminating than other factors: once a dog survives the early postoperative period, long-term outcomes after OMVR are similar across stages B2, C, and D (7).

#### 6.2.2 Mitral valve anatomy

Anatomic features that disqualify a dog from V-Clamp but not from OMVR include deep leaflet clefts, markedly thickened leaflets, significant leaflet flail (particularly posterior), inadequate leaflet coaptation depth, and mitral annular AP diameter greater than approximately 20 mm at end-systole or less than approximately 14 mm (9, 10). These features can be identified on preprocedural TEE or, with reasonable accuracy, on high-quality transthoracic echocardiography (9, 10). Preoperative imaging focused on these features is a prerequisite for V-Clamp candidacy assessment.

#### 6.2.3 Body size and mitral annular dimension

OMVR has been reported in dogs as small as 1.5 to 2 kg; however, published cohorts do not report outcomes separately for the sub-2 kg subgroup, so candidacy at this lower extreme currently rests on case-level rather than cohort-level evidence (6, 7). For V-Clamp, the operative constraint is not body weight but the mid-systolic anterior–posterior mitral annular diameter, which must accommodate the smallest (14 mm) device; body weight is only an approximate surrogate that varies with breed conformation, and annular diameter measured on preprocedural imaging should therefore govern candidacy rather than weight alone (9, 10). Very small dogs whose annular dimensions fall below the device range are more likely to be OMVR candidates if they are surgical candidates at all. Cardiopulmonary bypass in very small dogs also carries specific considerations, including a high circuit priming volume relative to circulating blood volume with consequent haemodilution and transfusion requirement, constraints on cannula sizing, and demanding thermal management, all of which contribute to the concentration of OMVR at high-volume centres (21, 27).

#### 6.2.4 Comorbidities

Significant pulmonary hypertension, advanced kidney disease, severe tracheal collapse, significant pre-existing neurologic disease, and uncontrolled systemic illness each independently increase perioperative risk. Published outcome data specifically support PH (TRV > 3.7 m/s) as a late-mortality modifier after OMVR and identify concurrent comorbid illness as a major contributor to adverse V-Clamp outcomes in at least one cohort (7, 10, 17). Comorbidity burden should be assessed formally before surgical referral.

#### 6.2.5 Center availability and experience

Given the geographic concentration of both techniques and the documented learning-curve effects, the realistic choice set for many patients is constrained by what centers are accessible. Referral to a center that has performed fewer than 50 cases of the planned procedure is not contraindicated, but should prompt explicit consideration of published outcome ranges at lower-experience centers during owner counseling (9, 10, 37).

### 6.3 Patient archetypes

No single surgical procedure is appropriate for all MMVD patients who progress beyond optimal medical management. Anatomic, clinical, and access factors define four broad archetypes among dogs whose owners are pursuing advanced care: dogs best suited to OMVR, dogs best suited to V-Clamp, dogs for whom either procedure is reasonable, and dogs better served by continued medical management. Recognizing all four as legitimate outcomes of the framework, rather than treating the surgical options as a hierarchy with medical management at the bottom, is central to honest counseling.

#### 6.3.1 The straightforward OMVR candidate

A small-breed dog of 2.5 to 6 kg in stage C on optimal medical therapy, with leaflet anatomy that includes a stretched annulus, elongated or ruptured chordae, and no markedly thickened leaflets, no significant flail at a disqualifying location, and no major comorbidities. Pulmonary hypertension, if present, is mild (TRV < 3.4 m/s). This is the classic published OMVR cohort profile, with expected discharge survival in the 95 to 96% range (7, 22). OMVR referral to an experienced surgical center is the most evidence-supported recommendation.

#### 6.3.2 The straightforward V-Clamp candidate

A small-breed dog of approximately 5 to 10 kg with stage B2 or C disease, favorable leaflet anatomy (adequate leaflet length, absence of deep clefts, AP diameter suitable for clamp sizing), limited or no comorbidities, and geographic access to a V-Clamp program with experience in the field. V-Clamp at an experienced center is reasonable, with the expectation of partial rather than complete MR reduction and the need for continued echocardiographic follow-up (9, 36).

#### 6.3.3 The borderline or high-risk candidate

A dog with stage D disease, significant PH, advanced age, multiple comorbidities, or anatomy marginal for V-Clamp. For these dogs, neither surgical option has clearly favorable evidence. OMVR has the broader anatomic and comorbidity reach but higher perioperative burden; V-Clamp is less invasive but has demonstrated worse outcomes in cohorts with high comorbidity prevalence. Decisions for these dogs require explicit weighing of published outcome ranges against the individual patient’s circumstances, with serious consideration of continued medical management as an equally reasonable path (10, 17).

#### 6.3.4 The dog better served by medical management alone

A dog in stage C or D with severe comorbidities that make general anesthesia genuinely prohibitive, with combined pre- and postcapillary pulmonary hypertension unlikely to resolve with surgical correction, or with owner priorities or resources that do not support surgical referral (10, 17). Optimal medical therapy delivered consistently, with attention to quality of life, is an honest and defensible choice and should not be framed as a failure of care. Especially in older dogs and in dogs with multiple comorbidities, conservative medical management is appropriately framed as a therapeutic option in its own right rather than as a failure to intervene.

### 6.4 Owner counseling

Communicating with owners about surgical options is a clinical skill informed by, but not reducible to, the published literature. Two considerations specific to this evolving field are worth highlighting. First, outcomes for both OMVR and V-Clamp vary across centers in ways that reflect cumulative team experience, selection discipline, and learning-curve effects (7, 9, 10, 37). Transparency about a referring center’s specific experience is therefore a meaningful component of the conversation for either procedure, particularly during early-experience program phases. Second, neither OMVR nor V-Clamp fully eliminates MR in all cases or guarantees long-term freedom from progression, and in some patients optimal medical management remains a clinically reasonable path. The framework presented in Sections 6.2 and 6.3 is intended to support these conversations rather than to substitute for them.

Beyond these two points, genuinely shared decision-making in this setting depends on several further elements that the framework is designed to surface rather than resolve. Cost and geographic access are legitimate determinants of the realistic choice set, not peripheral considerations, and are best raised early (Step 1) rather than after a family has committed to a referral pathway. Where a prospective referral centre sits on its own learning curve is itself material information for consent, particularly for V-Clamp, where every programme remains within its learning curve. Finally, the recommendation should be aligned with the individual owner’s priorities and the patient’s clinical window, so that continued medical management, when chosen for a dog in which anaesthetic risk or fixed pulmonary vascular disease makes intervention inadvisable, is presented as a positive and legitimate decision rather than as a withdrawal of care.

### 6.5 Center capability, center capacity, and the patient’s clinical window

The selection criteria in Section 6.2 emphasize center experience as a meaningful variable, and the Lee 2025 cohort confirms that early-program V-Clamp experience is associated with real perioperative mortality (10). At the same time, an exclusive emphasis on experience risks creating a clinical impasse: top-tier programs in both OMVR and V-Clamp routinely operate with multi-month waiting lists or temporarily suspended intake due to capacity constraints, and a stage C or D dog whose clinical trajectory does not permit such a wait may be left with continued medical management as the only feasible option even when capable, less-experienced centers exist with available capacity.[B.A.Maran, personal communication, May 2026] The honest framing of this tension is therefore not “top-tier center versus medical management” but “which center has both the capability and the capacity to provide care within the patient’s clinical window.” For a dog who can wait, referral to a high-volume program is the most evidence-supported choice. For a dog whose clinical decompensation does not permit waiting, a less-experienced center with available capacity and demonstrated procedural competence may be the appropriate referral, with owners counseled explicitly about the center’s specific experience and the published outcome range at comparable program-maturity phases. Both OMVR and V-Clamp experience this dichotomy; both procedures require ongoing global accumulation of cases for the field to mature, which in turn requires that referrals continue to flow to programs across the experience spectrum, not only to the most established ones. The framework presented here is therefore deliberately constructed to accommodate center capability, center capacity, and patient timing as joint inputs to the referral decision rather than to privilege any single one. Centre capability and capacity assessed against the patient’s clinical window are represented explicitly within Step 5 of the framework (Figure 1; Table 4), so that this dimension enters clinical reasoning directly rather than remaining a standalone consideration.

## 7 Discussion, future directions, and conclusion

### 7.1 Limitations of the current evidence base

The synthesis presented in this review rests on an evidence base with several important limitations. First, no randomized controlled trial has compared OMVR, V-Clamp, or medical management with one another in dogs with MMVD; outcome comparisons across techniques therefore reflect the populations selected by individual centers and may not generalize. Second, OMVR outcome data are heavily dominated by a single high-volume center, and V-Clamp outcome data, although recently extended to include multi-year follow-up at one institution, still derive from a small number of published cohorts; generalizability to community practice is uncertain. Third, methods for quantifying residual mitral regurgitation in dogs after intervention are not yet standardized, complicating direct outcome comparisons. Fourth, the framework proposed in Section 6 has not been validated prospectively, and its predictions should be treated as hypothesis-generating rather than as established clinical guidance (3, 7, 9, 10, 24, 36). Fifth, this manuscript is a literature synthesis rather than a report grounded in primary operator experience with V-Clamp TEER, and the authors therefore cannot speak to procedural decision-making, intraoperative challenges, or operator learning curves with the granularity that would come from active program experience; readers seeking practical procedural guidance should consult primary V-Clamp operators directly.

### 7.2 Future directions

Several priorities emerge for the next phase of canine mitral valve surgery research. Prospective multicenter cohort studies of both OMVR and V-Clamp, with standardized echocardiographic endpoints and follow-up beyond 12 months, would address the dominant evidence gap. A specific reporting priority within these studies is standardization of residual MR quantification using regurgitant fraction or an analogous quantitative metric across both OMVR and V-Clamp cohorts, which would resolve the methodological asymmetry between the two literatures discussed in Section 3.2. Validated anatomic eligibility criteria for V-Clamp, derived from prospective comparison of patients in whom procedures succeeded versus failed, would refine selection. A canine analog of the human COAPT trial design, comparing TEER plus medical management to medical management alone, would directly establish whether intervention improves survival in dogs unsuitable for OMVR. Additional priorities identified by this review include standardisation of postoperative pharmacotherapy and antithrombotic protocols after both procedures; comparative reporting of new-onset and persistent atrial fibrillation, spontaneous conversion probability, and anticoagulation strategy after each technique; and standardised reporting of operative time, cross-clamp duration, and quantitative residual MR across the OMVR technical variants compared in Table 5. Transcatheter mitral valve replacement, currently in early experimental development, may eventually expand surgical options for patients ineligible for repair (9, 34, 35). In addition, the canine transcatheter mitral valve repair market is expected to broaden beyond V-Clamp; a second canine TEER device has been publicly announced by NaCure (V-TEER) but is not yet reported in the peer-reviewed clinical literature at the time of writing. This device has been described only in company communications and meeting presentations; no peer-reviewed clinical data have been published, and it is noted here solely to indicate an emerging development rather than as evidence of performance. The V-TEER device has been presented in NaCure Medical public communications, including the company’s verified Instagram and Facebook channels (posts dated March 18, March 22, and April 28, 2026) and at multiple international veterinary cardiology meetings (41). The emergence of additional devices reflects continued client and clinician demand for less-invasive options and reinforces the importance of standardized outcome reporting across devices and centers as the field expands.

**Table 5:** Comparison of principal OMVR technical variants.

Variant (reference)	Chordal method	Annuloplasty method	Largest published cohort	Operative / CPB time	Residual MR reporting	Long-term durability data
Standard Uechi technique (6, 7, 21)	Multiple ePTFE artificial chordae	Circumferential suture annuloplasty	1,019 dogs (7)	Median CPB ~ 165 min (17); total anesthesia ~7 h	Qualitative (grade/jet-area ratio); quantitative RF not reported	Longest available; events tracked to ~1,400 days (7)
Loop-in-loop (22)	Pre-measured ePTFE loops on a papillary strut	Circumferential suture annuloplasty	55 dogs (22)	Not reported separately	Qualitative; reverse-remodelling indices to 24 months	Reverse remodelling sustained to 24 months (22)
Chayagasaka technique (11)	Artificial chordae	Semicircular annuloplasty + direct scallop-suture valvuloplasty	Case series (11)	Not reported	Qualitative	Not reported
Isayama anchoring modification (23)	Novel artificial-chordae anchoring method	As per base technique	Technique report (23)	Not reported	Qualitative	Not reported

CPB, cardiopulmonary bypass; ePTFE, expanded polytetrafluoroethylene; MR, mitral regurgitation; OMVR, open mitral valve repair; RF, regurgitant fraction. Several cells are populated as “not reported” because operative time, cross-clamp duration, and quantitative residual MR are inconsistently reported across the variant literatures, and no head-to-head comparison of variants has been performed. Standardised procedural reporting across variants is identified as a research priority in Section 7.2.

## 8 Conclusion

Surgical management of MMVD in dogs is a rapidly evolving field with two clinically established options of differing maturity, invasiveness, and evidence base. The framework proposed in this review, applied with attention to individual patient circumstances and to evolving published evidence, is intended to support honest clinical decision-making and transparent owner counseling.
